# Long Non-Coding RNA GRIK1-AS1 Inhibits the Proliferation and Invasion of Gastric Cancer Cells by Regulating the miR-375/IFIT2 Axis

**DOI:** 10.3389/fonc.2021.754834

**Published:** 2021-09-30

**Authors:** Qi Zhou, Yuan Li, Lujun Chen, Xiao Zheng, Tianwei Jiang, Juan Liu, Yuanyuan Fu, Liping Guan, Jingfang Ju, Changping Wu

**Affiliations:** ^1^ Department of Oncology, The Third Affiliated Hospital of Soochow University, Changzhou, China; ^2^ Department of Tumor Biological Treatment, The Third Affiliated Hospital of Soochow University, Changzhou, China; ^3^ Jiangsu Engineering Research Center for Tumor Immunotherapy, Changzhou, China; ^4^ Institute of Cell Therapy, Soochow University, Changzhou, China; ^5^ Department of Neurosurgery, The Third Affiliated Hospital of Soochow University, Changzhou, China; ^6^ Translational Research Laboratory, Department of Pathology, Stony Brook University, Stony Brook, NY, United States

**Keywords:** long non-coding RNA, lncRNA GRIK1-AS1, miR-375, IFIT2, gastric cancer

## Abstract

Long non-coding RNAs (lncRNAs) play important roles in various biological processes and human diseases, including cancer. In this study, we demonstrated a regulatory relationship between lncRNA GRIK1-AS1 and miR-375/IFIT2 axis in gastric cancer. Our results show a decreased expression of GRIK1-AS1 in gastric cancer tissues compared to adjacent normal gastric tissues. Gastric cell lines also have reduced levels of GRIK1-AS1 compared to gastric epithelial cell line GES-1. Ectopic expression of GRIK1-AS1 in gastric cancer cell lines significantly inhibits cellular viability, migration, and invasion. RNA-pull down and the luciferase activity assays show that GRIK1-AS1 mainly interacts specifically with miR-375. We further demonstrate a negatively regulatory relationship between lncRNA GRIK1-AS1 and miR-375. We discovered that IFIT2 was one of the direct key downstream target genes of miR-375, and established the important role of the GRIK1-AS1/miR-375/IFIT2 axis in the progression of gastric cancer. Taken together, our results revealed a novel mechanism of GRIK1-AS1 as a sponge to miR-375 that impacts gastric cancer progression *via* modulating target mRNA IFIT2 translation, and as a result, opens a new strategy to future GRIK1-AS1 based therapeutic development.

## Introduction

Gastric cancer is the second most common cancer-related mortality after lung cancer worldwide (1–3). Despite the extensive research and development effort, the prognosis is still dismal with an average five-year survival rate of less than 20% (4). The majority of cases are diagnosed at the advanced disease stage with distant metastasis (5, 6). Therefore, there is an urgent need to identify novel therapeutics for gastric cancer patients, and long non-coding RNAs (lncRNAs) are one of these molecules with such potential.

LncRNAs are a class of non-coding RNA with lengths exceeding 200 nucleotides (7). LncRNAs have diverse functions which can act as guides, decoys, scaffolds, and tethers of other biological molecules (8–10). Besides, lncRNAs can competitively sponge miRNAs from their target mRNA transcripts (11). LncRNAs participate in various biological processes including cell growth, migration, apoptosis, differentiation, and stem cell pluripotency (12, 13). Growing evidence supports the critical functions of lncRNAs in human cancers. Previous studies have identified several deregulated lncRNA expression in gastric cancer with critical functions (14). For instance, SNHG12 was demonstrated to regulate cancer progression by sponging miR-320 (15). HOTAIR could target miR-126 to activate PI3K/AKT/MRP1 signal pathway to promote cisplatin resistance (16). The lncRNA GRIK1-AS1 (Glutamate Ionotropic Receptor Kainate Type Subunit 1-Antisense RNA1, ENSG00000174680) is located at chr21q21.3, and has been previously reported to be down-regulated in gastric cancer tissues (14). However, the biological function of lncRNA GRIK1-AS1 in gastric cancer progression remains elusive.

In this study, we found that lncRNA GRIK1-AS1 was down-regulated in gastric cancer tissues and cell lines. Moreover, over-expression of GRIK1-AS1 significantly inhibited gastric cancer cell proliferation and invasion. Based on the bioinformatics analysis, we discovered that GRIK1-AS1 may directly bind to miR-375 as a sponge to increase its target IFIT2 protein level. Our direct experimental evidence based on a series of rescue experiments and RNA-pull down assays directly confirmed the regulatory relationship between GRIK1-AS1 and miR-375/IFIT2 axis using gastric cancer cell lines. The functional significance of the GRIK1-AS1/miR-375/IFIT2 axis in gastric cancer progression and metastasis supports the future development of GRIK1-AS1 based therapeutic development.

## Materials and Methods

### Human Gastric Cancer Tissue Specimens

Gastric cancer tissues and paired adjacent normal gastric epithelial tissues were obtained from the Third Affiliated Hospital of Soochow University from 2003 to 2015. Tissue specimens were obtained through needle puncture and stored in liquid nitrogen at -80°C. All experiments using the human tissues were conducted under the approval of the Third Affiliated Hospital of Soochow University. All patients gave informed consent.

### Cell Lines and Cell Culture

All cells in this study were obtained from the Chinese Academy of Sciences, Shanghai Institutes for Biological Sciences. HEK293 cells were cultured in DMEM (Gibco, Melbourne, Australia) supplemented with 10% fetal bovine serum (FBS, Gibco, Melbourne, Australia). Gastric epithelial cell GES-1 and gastric cancer cells AGS, SGC-7901, and HGC-27 were cultured in RPMI 1640 (Gibco, Melbourne, Australia) supplemented with 10% FBS.

### Expression Plasmids and siRNA

The full-length human lncRNA GRIK1-AS1 cDNA was cloned into pLVX-IRES-Puro vector (Clontech, CA, USA) to generate GRIK1-AS1 expression plasmids. The siRNA targeting IFIT2 was purchased from GenePharma (Shanghai, China).

### Cell Transfection

siRNA duplexes (100nM), miR-375 mimic, and corresponding negative control (NC) oligonucleotides were transfected into cells mediated by Lipofectamine 3000 (Thermo Fisher, CA, USA) according to manufacturer’s instructions.

### Lentivirus Production and Infection

To establish individual stable cells, lentivirus was used. Lentiviral particles in cell culture supernatant were harvested at 72h after lentivirus transfection into HEK293 cells. The AGS and SGC-7901 cells were then infected with lentivirus. Stably transfected cells were selected by puromycin (2µg/mL, Sigma-Aldrich, MO, USA) for 1 week at 48h after lentiviral infection.

### Cell Proliferation Assays

Cell proliferation assay was performed using CellTiter 96^®^ Non-Radioactive Cell Proliferation Assay (MTT) kit (Promega) according to the manufacturer’s instructions. Cells (1×10^4^cells/mL) were seeded into a 96-well plate (100 µl/well) and cultured in an incubator with 5% CO_2_ at 37°C. Each well was added with 10 µl MTS solution and then incubated at 37°C for 2h. The spectrophotometric absorbance at 590 nm was measured for each sample. All the experiments were repeated 3 times in triplicates.

### Transwell Assay

The cell invasion ability was measured by Matrigel-coated transwell assay, in which transwell chambers (8-μm pore size; Corning) were applied. Cells (1×10^5^) were seeded into the upper chamber with serum-free RPMI 1640 medium, and RPMI 1640 medium supplemented with 20% fetal bovine serum was added into the lower chamber. After 48h incubation, cells on the top surface of filters in the upper chambers were cleaned up gently with a cotton swab. Then, the filters were fixed in 4% PFA for 15 min and stained with 0.1% crystal violet for 10 min. After washed by PBS for 3 times, cells migrated across the filters were imaged and the number of invaded cells was calculated by counting five random views under a microscope. The experiment was performed in triplicates and repeated 3 times.

### Wound Healing Assay

A wound healing assay was performed to determine the cell migration capability. Cells were seeded in 6-well plates. When the cells were about 90% confluent, the artificial wounds were cut using a 200-ul pipette tube. After 24h, the wound closure was observed and imaged under a microscope. The migration distance was indicated by the fraction of cell coverage across the line. Triplicates were required for each experiment.

### RNA Isolation and Real-Time PCR

Total RNA was extracted using TRIzol according to the manufacturer’s instructions. First-strand cDNA was synthesized using Superscript II (Invitrogen) and 1μg of total RNA was used in each cDNA synthesis reaction. SYBR green Universal Master Mix reagent (Roche) and primer mixtures were used for the real-time qPCR. GAPDH was used as the internal reference for mRNAs.

The miRNAs expression level was measured using the All-in-One™miRNAqRT-PCR Detection Kit (GeneCopoeia)according to the manufacturer’s instructions.U6 small RNA was used as the reference. The primers used for real-time qPCR were as follows: LncRNA GRIK1-AS1, forward: 5’-ATGGAGGATGCAGCAAAAGGG-3’; reverse: 5’-TTCTGTGTCCTGGTTGTTTCTC-3’;IFIT2, forward: 5’-AAGCACCTCAAAGGGCAAAAC-3’; reverse: 5’-TCGGCCCATGTGATAGTAGAC-3’;GAPDH, forward: 5’-CTGGGCTACACTGAGCACC-3’; reverse: 5’-AAGTGGTCGTTGAGGGCAATG-3’;U6, forward: 5’-CTCGCTTCGGCAGCACA-3’; reverse: 5’-AACGCTTCACGAATTTGCGT-3’.

### Nuclear Mass Separation

SurePrep™ Nuclear or Cytoplasmic RNA Purification Kit (Fisher BioReagents) was applied to isolate nuclear and cytoplasmic fractions according to the manufacturer’s instructions. RNA levels of lncRNA GRIK1-AS1, the nuclear control RNU6-1, and the cytoplasmic control GAPDH were analyzed by real-time qPCR.

### RNA-Fluorescence *In Situ* Hybridization

Cells were fixed in 4% PFA for 15 min and then permeabilized by 0.5% TritonX-100 for 15 min at 4°C.0.5% TritonX-100 was used to permeabilize the cells for 15 min at 4°C. Digoxigenin (DIG) labeled GRIK1-AS1 probe or control probe mix haswas performed to incubate cells for 4h at 55°C. After 2xsaline-sodium citrate briefly washing 3 times with 5 min every time, signals were detected by Horseradish peroxidase (HRP)-conjugated anti-DIG secondary antibodies (Jackson). DAPI was used to counterstain nuclear. A con-focal laser scanning microscope was used for images.

### Western Blotting Analyses

Total proteins were extracted and separated using SDA-PAGE gels. IFIT2 antibody (Abcam MA, USA) was used at a concentration of 1:2000. GAPDH antibody (Santa Cruz Biotechnology, 1:2000, CA. USA) was used as a loading control.

### Reporter Vector Construction and Luciferase Reporter Assays

GRIK1-AS1-WT luciferase reporter vector was conducted by cloning GRIK1-AS1 cDNA containing predictive miR-375 binding site into the pmirGLO Dual-Luciferase miRNA Target Expression Vector (Promega, WA, USA). GRIK1-AS1-Mut vector was generated by insertion of mutant GRIK1-AS1 containing point mutations in the miR-375 binding site. Likewise, wild-type and mutant IFIT2 3’-UTR fragments were cloned into pmirGLO vector to conduct the IFIT2 3’UTR-WT and IFIT2 3’UTR-Mut luciferase reporter vectors. The miR-375 or miR-NC was co-transfected with the reporter vector into HEK293 cells using Lipofectamine 3000 (Invitrogen). The luciferase activity was measured at 48h after transfection using the Dual-Luciferase Reporter Assay System (Promega) according to the manufacturer’s instructions. Triplicates were required for each experiment.

### RNA-Pull Down

3’-end biotinylated miR-375, miR-375-Mut, or candidate miRNAs were transfected into cells at a final concentration of 20 nmol/L. After 24h, cells were harvested and incubated in the cell lysate with streptavidin-coated magnetic beads (Ambion, Life Technologies). The biotin-coupled RNA complex was pulled down and analyses of the abundance of GRIK1-AS1 were conducted by real-time qPCR.

### Statistical Analysis

All experiments were performed using 3 independent repeated experiments with cells. GraphPad Prism 8.0 was applied for statistical analyses. Data in all figures are presented as the mean ± SEM. Statistical significance was determined by Student’s *t* test, One-way ANOVA and Two-way ANOVA. For all statistical tests, the 0.05 level of confidence (2-sided) was accepted for statistical significance.

## Results

### Decreased LncRNA GRIK1-AS1 Expression in Gastric Cancer Tissues

**Figure 1:**
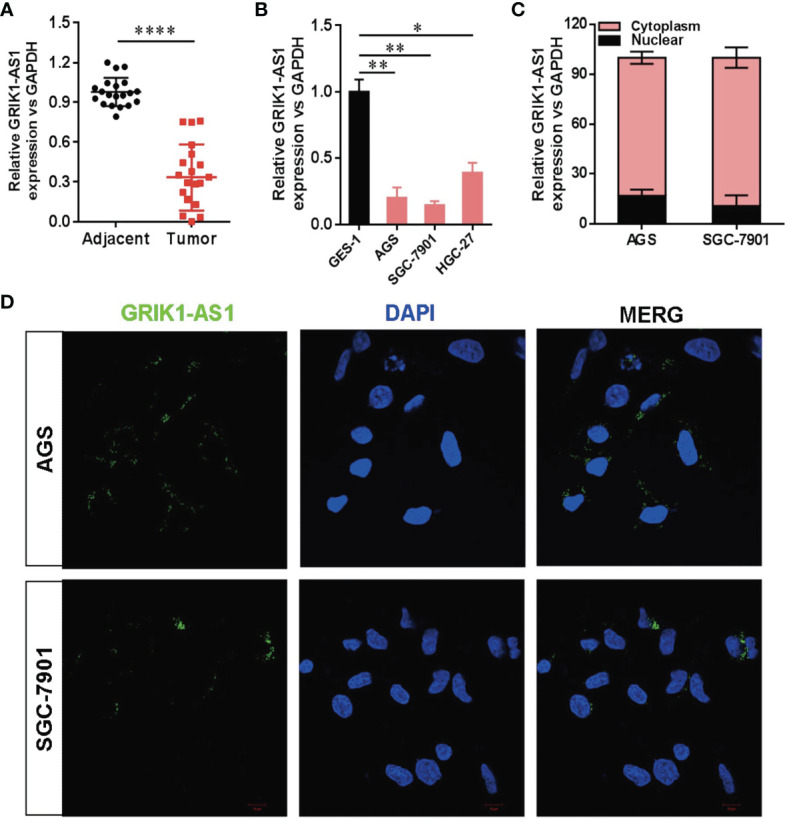
Decreased LncRNA GRIK1-AS1 expression in gastric cancer.**(A)**. Real-time qPCR analysis of GRIK1-AS1 expression in gastric cancer tissues and adjacent normal tissues. **(B)**. Real-time qPCR analysis of GRIK1-AS1 expression in gastric epithelial cell GES-1 and gastric cancer cells AGS, SGC-7901 and HGC-27. **(C)**. The sub-cellular localization of GRIK1-AS1 in gastric cancer cells (AGS and SGC-7901) was determined by the nuclear/cytoplasm distribution analyses and RNA-FISH assays. **(D)**. Fluorescence *in situ* hybridization (FISH) analysis indicates the cytoplasmic localization of GRIK1-AS1 in AGS and SGC-7901 cells. *P < 0.05, **P < 0.01, ****P < 0.0001.

To determine the potential disease relevance of GRIK1-AS1 in gastric cancer, the real-time qPCR analysis was used to quantify the expression of GRIK1-AS1 in both gastric cancer tissues and cell lines. As indicated in **Figure 1A**, GRIK1-AS1 expression was significantly decreased (*P*<0.005) in tumors compared with adjacent normal tissues (**Figure 1A**). Likewise, the gastric cancer cells (AGS, SGC-7901, HGC-27) showed a lower expression level of GRIK1-AS1 than gastric epithelial cell GES-1 (**Figure 1B**). These results indicate that decreased GRIK1-AS1 expression might be associated with the development of gastric cancer. Next, we aimed to explore the molecular basis of GRIK1-AS1 functioning as a tumor-suppressor in gastric cancer. First, the sub-cellular localization of GRIK1-AS1 in gastric cancer cells (AGS and SGC-7901) was determined by the nuclear/cytoplasm distribution analyses and RNA-FISH assays. Results showed that GRIK1-AS1 was mainly located in the cytoplasm (**Figures 1C, D**).

### LncRNA GRIK1-AS1 Impairs Cancer Progression *In Vitro*

**Figure 2:**
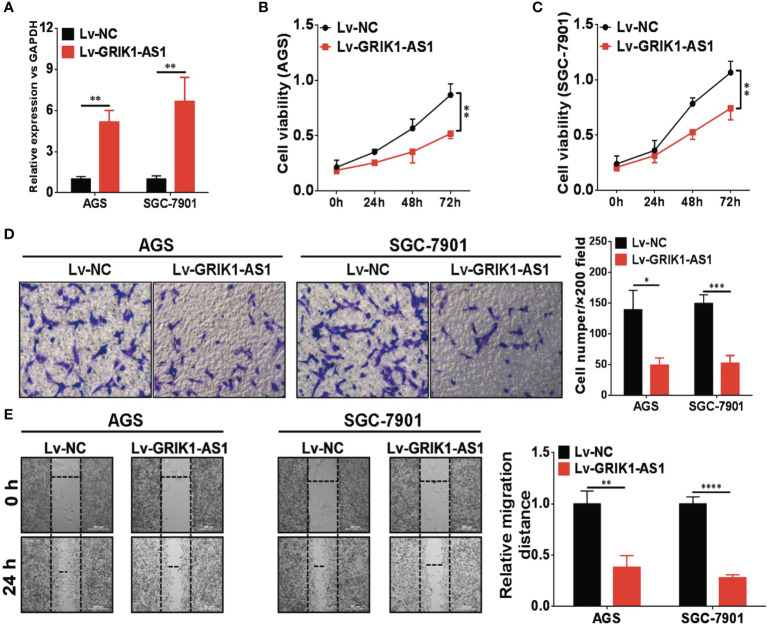
LncRNA GRIK1-AS1 inhibits cell proliferation and invasion *in vitro*. **(A)** Real-time qPCR shows the expression level of GRIK1-AS1 in gastric cancer cells AGS and SGC-7901 and its over-expression. **(B, C)** Cell proliferation assays of GRIK1-AS1 over-expressed AGS and SGC-7901 cells. **(D)** Invasion capability analysis of GRIK1-AS1 over-expressed AGS and SGC-7901 cells by transwell assays. **(E)** Migration ability analysis of GRIK1-AS1 over-expressed AGS and SGC-7901 cells by wound healing assays. *P < 0.05, **P < 0.01, ***P < 0.005, ****P < 0.0001.

To investigate the potential functional significance of GRIK1-AS1 in gastric cancer, we ectopically over-expressed GRIK1-AS1 in gastric cancer cells AGS and SGC-7901 (**Figure 2A**). We found that restoration of GRIK1-AS1 significantly attenuated cancer cell proliferation (**Figures 2B, C**). Moreover, the increased GRIK1-AS1 expression significantly suppressed cancer cell invasion and migration abilities (**Figures 2D, E**). These results support the potential tumor suppressor role of lncRNA GRIK1-AS1 in gastric cancer.

### LncRNA GRIK1-AS1 Functions as a ceRNA for miR-375

**Figure 3:**
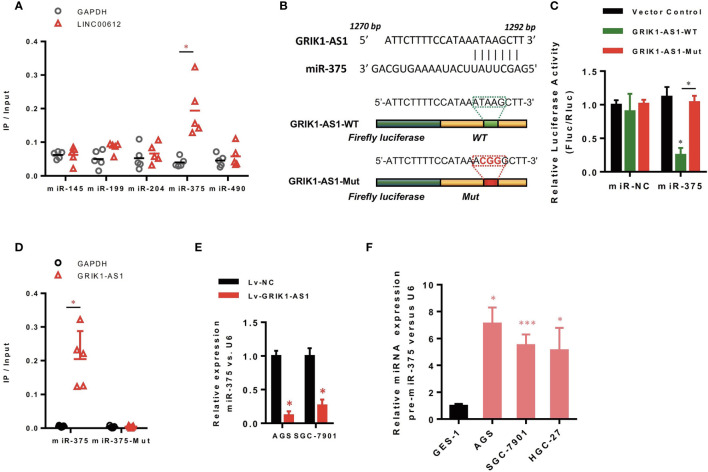
LncRNA GRIK1-AS1 functions as the competitive sponges for miR-375. **(A)** RNA-pull down was performed to screen out miR-375 and RNA level of GRIK1-AS1 was quantified by real-time qPCR. **(B)** A schematic diagram showing the putative miR-375 binding sites with GRIK1-AS1. **(C)**GRIK1-AS1-WT and GRIK1-AS1-Mut luciferase reporter vectors were co-transfected with miR-375 or miR-NC and then subjected to luciferase activity analyses.**(D)** RNA-pull down was performed to confirm the putative miR-375 binding sites with GRIK1-AS1. **(E)** Real-time qPCR analysis of miR-375 levels in GRIK1-AS1 over-expressed AGS and SGC-7901 cells. **(F)** Real-time qPCR analysis of miR-375 level in gastric epithelial cell GES-1 and gastric cancer cells AGS, SGC-7901 and HGC-27. *P < 0.05, ***P < 0.005. Scale bars: 50µm.

Increasing evidence demonstrated that lncRNAs located in the cytoplasm can function as the competitive endogenous RNAs (ceRNAs) for miRNAs, competitively sponging miRNAs to inhibit its regulatory effect on target mRNAs. We then analyzed the miRNA-recognition sequence of GRIK1-AS1 *via* bioinformatics tools (Star Base, Target Scan, miRcode) and found a list of miRNAs with the complementary sequence to GRIK1-AS1. Using RNA-pull down assays, we screened out the most interacted miRNA. Results showed that GRIK1-AS1 mainly interacted with miR-375 instead of other miRNA candidates (**Figure 3A**). And the luciferase activity assays were performed to further confirm the regulation of GRIK1-AS1 to miR-375 (**Figures 3B, C**). GRIK1-AS1 cDNA containing predicted miR-375 binding site was cloned into luciferase reporter vectors (GRIK1-AS1-WT) and then co-transfected with miR-NC or miR-375. Results showed that the luciferase activity was significantly decreased when GRIK1-AS1-WT was co-transfected with miR-375. Meanwhile, the GRIK1-AS1-Mut luciferase reporter vector was also conducted with a mutation in its miR-375 binding site. Co-transfection with GRIK1-AS1-Mut and miR-375 did not reduce the luciferase activity (**Figure 3C**). RNA-pull down results confirmed that GRIK1-AS interacted with wild-type miR-375 instead of mutant miR-375 (**Figure 3D**).

**Figure 4:**
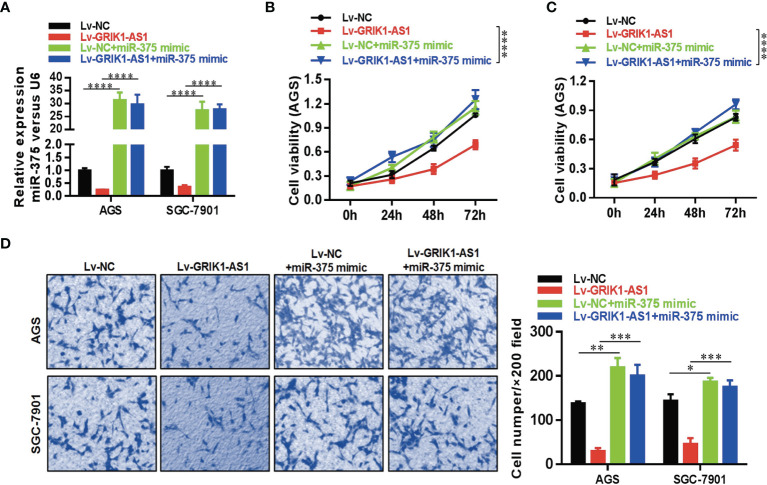
miR-375 over-expression reverses the suppressed cell proliferation and invasion capability caused by GRIK1-AS1 over-expression. **(A)** Real-time qPCR analysis of miR-375 level in control and GRIK1-AS1 over-expressed AGS and SGC-7901 cells with or without miR-375 over-expression. **(B, C)** Cell proliferation assays of control and GRIK1-AS1 over-expressed AGS and SGC-7901 cells with or without miR-375 over-expression. **(D)** Trans-well assays of control and GRIK1-AS1 over-expressed AGS and SGC-7901 cells with or without miR-375 over-expression. *P < 0.05, **P < 0.01, ***P < 0.005, ****P < 0.0001.

To further support the direct regulatory relationship between GRIK1-AS1 and miR-375 in gastric cancer cells. Real-time qPCR analyses over-expression of GRIK1-AS1 in AGS and SGC-7901 cells led to a significant down-regulation of miR-375 (**Figure 3E**), which was exactly the opposite from the expression pattern of GRIK1-AS1 (**Figure 1B**). Meanwhile, pre-miR-375 displayed a markedly higher expression level in gastric cancer cells than in gastric epithelial cells (**Figure 3F**). Moreover, miR-375 mimic reversed the suppressed cell proliferation (**Figures 4A–C**) and invasion (**Figure 4D**) capability caused by the over-expression of GRIK1-AS1 in gastric cancer cells. These results, taken together, demonstrated a negatively regulatory relationship between lncRNA GRIK1-AS1 and miR-375.

### LncRNA GRIK1-AS1 Modulates miR-375/IFIT2 Axis in Gastric Cancer

**Figure 5:**
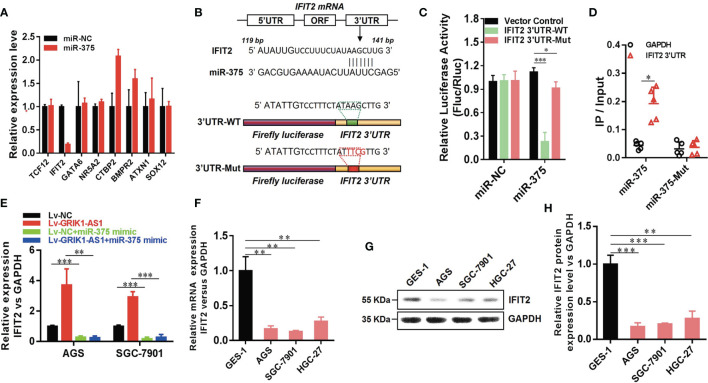
LncRNA GRIK1-AS1 regulates miR-375 to modulate IFIT2 in gastric cancer. **(A)**. Real-time qPCR was performed to screen out IFIT2 as miR-375 target gene. **(B)** A schematic diagram showing the putative miR-375 binding sites with IFIT2 3’UTR. **(C)**IFIT2 3’UTR-WT and IFIT2 3’UTR-Mut luciferase reporter vectors were co-transfected with miR-375 or miR-NC and then subjected to luciferase activity analysis. **(D)** RNA-pull down was performed to confirm the putative miR-375 binding sites with IFIT2 3’UTR. **(E)** Real-time qPCR analysis of IFIT2 expression level in control and GRIK1-AS1 over-expressed AGS and SGC-7901 cells with or without miR-375 over-expression. **(F)** Real-time qPCR analysis of IFIT2 expression level in gastric epithelial cell GES-1 and gastric cancer cells AGS, SGC-7901 and HGC-27. **(G, H)** Western blotting analysis of IFIT2 protein level in gastric epithelial cell GES-1 and gastric cancer cells AGS, SGC-7901 and HGC-27. *P < 0.05, **P < 0.01, ***P < 0.005.

Having demonstrated the functional significance of lncRNA GRIK1-AS1 in gastric cancer by impacting miR-375 expression, we began to identify the critical target of miR-375 in gastric cancer. Based on target analysis such as Target Scan, we found several target genes whose 3’-UTR contained miR-375 binding sites. miR-NC and miR-375 were separately transfected into HEK293 cells and real-time qPCR was performed to measure the expression level of these candidate targets. IFIT2 was successfully screened out as the direct downstream target gene of miR-375 (**Figure 5A**). Luciferase activity assays indicated that miR-375 significantly reduced the expression of wild-type IFIT2 3’UTR, while did not affect mutant IFIT2 3’UTR expression (**Figures 5B, C**). The results obtained from luciferase activity assays were further strengthened by RNA-pull down (**Figure 5D**). IFIT2 3’-UTR directly interacted with wild-type miR-375 instead of its mutant.

**Figure 6:**
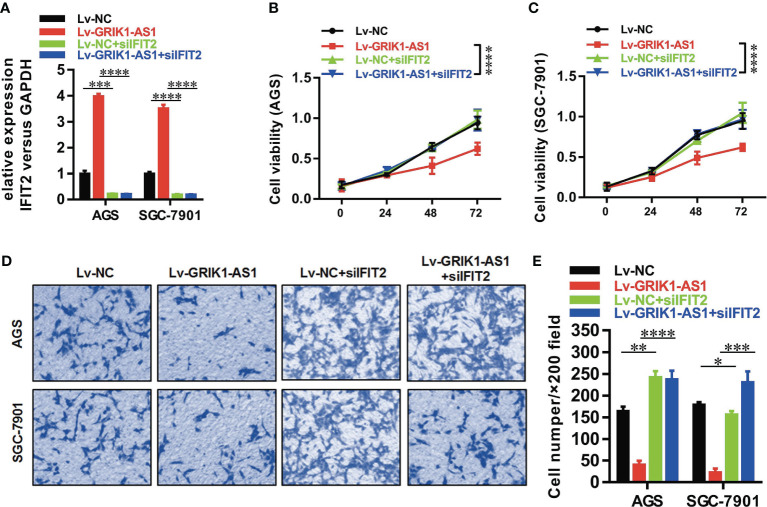
IFIT2 knockdown reverses the suppressed cell proliferation and invasion capability caused by GRIK1-AS1 over-expression.**(A)** Real-time qPCR analysis of IFIT2 expression level in control and GRIK1-AS1 over-expressed AGS and SGC-7901 cells with or without IFIT2 knockdown. **(B, C)** Cell proliferation assays of control and GRIK1-AS1 over-expressed AGS and SGC-7901 cells with or without IFIT2 knockdown. **(D, E)** Transwell assays of control and GRIK1-AS1 over-expressed AGS and SGC-7901 cells with or without IFIT2 knockdown. *P < 0.05, **P < 0.01, ***P < 0.005, ****P < 0.0001.

To better establish the relationship between GRIK1-AS1 and miR-375 target gene IFIT2, the IFIT2 expression level was measured in GRIK1-AS1 over-expressed AGS and SGC-7901 cells. As indicated in **Figure 5E**, IFIT2 expression was markedly up-regulated by GRIK1-AS1 over-expression, while miR-375 over-expression significantly down-regulated IFIT2 (**Figure 5E**). Real-time qPCR (**Figure 5F**) and western blotting (**Figures 5G, H**) analyses indicate a significant (AGS *vs* GES-1: *P*<0.005, SGC-7901 *vs* GES-1: *P*<0.005, HGC27 *vs* GES-1: *P*<0.01) lower expression level of IFIT2 in gastric cancer cells than in gastric epithelial cells. Additionally, IFIT2 knockdown reversed the suppressed cell proliferation and invasion capability caused by the over-expression of GRIK1-AS1 in AGS and SGC-7901 cells (**Figures 6A–E**). Together, these results corroborated that GRIK1-AS1/miR-375/IFIT2 axis is a novel regulatory mechanism in gastric cancer progression.

## Discussion

In this study, we discovered a novel regulatory mechanism of lncRNA GRIK1-AS1 with miR-375 and functional impact on gastric cancer through IFIT2 as the major target. Our *in vitro* experiments indicate that GRIK1-AS1 over-expression significantly impaired cancer progression, inhibiting cancer cell proliferation and invasion. These results support the tumor suppressor function of GRIK1-AS1 in gastric cancer. Previous studies revealed that many lncRNAs were dysregulated in gastric cancer, regulating cancer progression and metastasis. For instance, lncRNA H19, HOXA11-AS, and SNHG12 were all associated with gastric cancer progression (17, 18).

LncRNAs exert different functions depending on their distinct nuclear/cytoplasm distribution. The nuclear lncRNAs are involved in epigenetic or transcription regulation *via* chromosome modulation or interacting with nuclear proteins like transcription factors (8, 19), while the cytoplasmic lncRNAs function as ceRNAs for miRNAs (11). Therefore, to investigate the genetic mechanisms of lncRNA GRIK1-AS1 in gastric cancer, we first analyzed the nuclear/cytoplasm distribution of GRIK1-AS1 and found that it was mainly located in the cytoplasm. Using predictive bioinformatics tools and combined with RNA-pull down and luciferase reporter assays, we successfully screened out the miR-375 and its target gene IFIT2. Although the expression of pre-miR-375 was overexpressed in gastric cancer cell lines (**Figure 3F**), in contrast, the mature miR-375 was significantly down-regulated by GRIK1-AS1 in gastric cancer cells (**Figure 3E**). These results suggest that the interaction of GRIK1-AS1 with miR-375 caused degradation of mature miR-375 expression. Such mechanism of lncRNA triggered miRNA degradation has been demonstrated in the literature by others as targeted miRNA degradation. Functionally, the over-expression of miR-375 or knockdown of IFIT2 perfectly reversed the suppressed cell proliferation and invasive capability caused by GRIK1-AS1 over-expression in gastric cancer cells. These results revealed that the function of lncRNA GRIK1-AS1, at least in part, was relied on the miR-375/IFIT2 axis to exert its biological functions. Sponging functions of circular RNAs, circ-SERPINE2 and YWHAZ, have been reported in a recent study to influencing miR-375 function in gastric cancer (20). It seems that gastric cancer leverages multiple mechanisms of lncRNA and circular RNA to exert the function of modulating protein expression *via* influencing mRNA binding activity.

IFIT2 (IFN-induced protein with tetratricopeptide repeats 2), whose another name is IFN-stimulated gene 54 (ISG54), is highly responsive to IFN stimulation. IFIT2 has been demonstrated to perform important anti-cancer and anti-virus functions (21, 22). We have previously reported that decreased IFIT2 expression was found in gastric cancer tissues, and its expression level was significantly associated with tumor stage and postoperative prognoses of the patients (23). Tang et al. also found that lncRNA00364 could up-regulate IFIT2 *via* inhibiting STAT3 phosphorylation to repress hepatocellular carcinoma (24). Ohsugi et al. revealed that excessive Wnt/β-catenin signaling decreased IFIT2 expression to inhibit apoptosis in colorectal cancer (25). In our present study, we found that IFIT2, the miR-375 targeted gene, was decreased in gastric cancer by loss of lncRNA GRIK1-AS1 which competitively sponges miR-375. It is highly consistent with previous studies that IFIT2 is down-regulated in human cancers. Although it is likely that miR-375 also suppresses other potential important targets such as the Hippo pathway through direct targeting YAP1, TEAD4 and CTGF (26), JAK2 (27), and PDK1 or 14-3-3zeta (28), the mechanisms of reduced GRIK1-AS1, as well as the downstream of IFIT2 in gastric cancer merit further investigation.

In summary, we identify a novel regulatory mechanism of GRIK1-AS1 with miR-375 and its direct target IFIT2 in gastric cancer. The reduced expression of GRIK1-AS1 was responsible for gastric cancer progression by impacting proliferation, invasion, and metastasis. As a result, lncRNA GRIK-AS1 may act as a tumor suppressor in gastric cancer, and future efforts on modulating GRIK1-AS1 maybe a unique therapeutic strategy.

## Data Availability Statement

The raw data supporting the conclusions of this article will be made available by the authors, without undue reservation.

## Author Contributions

QZ, YL, LC, XZ, TJ, JL, LG, and YF performed experiments and did the data analysis. LC and CW performed statistical analysis. JJ and CW designed the study. All authors contributed to the article and approved the submitted version.

## Funding

This work was supported by grants from the National Natural Science Foundation of China (31570877, 31800745), the Key R&D Project of Science and Technology Department of Jiangsu Province (BE2016660, BE2018645), Changzhou High-Level Medical Talents Training Project (No. 2016CZBJ001), Scientific and Technological Support Program for Social Development of Changzhou Sci and Tech Bureau (Grant No. : CE20215042).

## Conflict of Interest

The authors declare that the research was conducted in the absence of any commercial or financial relationships that could be construed as a potential conflict of interest.

## Publisher’s Note

All claims expressed in this article are solely those of the authors and do not necessarily represent those of their affiliated organizations, or those of the publisher, the editors and the reviewers. Any product that may be evaluated in this article, or claim that may be made by its manufacturer, is not guaranteed or endorsed by the publisher.
